# Detection of tetracycline resistance genes and their diversity in *Escherichia coli* isolated from pig farm waste in Banten province, Indonesia

**DOI:** 10.14202/vetworld.2023.1907-1916

**Published:** 2023-09-21

**Authors:** Debby Fadhilah Pazra, Hadri Latif, Chaerul Basri, I. Wayan Teguh Wibawan, Puji Rahayu

**Affiliations:** 1 Animal Biomedical Science Study Program, School of Veterinary Medicine and Biomedical Sciences (SVMBS), IPB University, Bogor, Indonesia; 2 Bogor Agricultural Development Polytechnic, Bogor, Indonesia; 3 Division of Veterinary Public Health and Epidemiology, School of Veterinary Medicine and Biomedical Sciences (SVMBS), IPB University, Bogor, Indonesia; 4 Division of Medical Microbiology, School of Veterinary Medicine and Biomedical Sciences (SVMBS), IPB University, Bogor, Indonesia; 5 Quality Control Laboratory and Certification of Animal Products, Bogor, Indonesia

**Keywords:** *Escherichia coli*, pig farms, *tet* genes, tetracycline resistance, waste

## Abstract

**Background and Aim:**

Livestock waste in the form of feces and liquid represents an important reservoir of antibiotic resistance genes (ARGs). Because many ARGs can be horizontally transferred to other pathogens, livestock waste plays an essential role in the emergence and transmission of various ARGs in the environment. Therefore, this study aimed to detect and assess the diversity of *tet* genes in *Escherichia coli* isolated from pig farm waste in Banten province, Indonesia.

**Materials and Methods:**

Solid waste (feces) and wastewater were collected from 44 pig farms in Banten province. The isolation and identification of *E. coli* referred to the Global Tricycle Surveillance extended-spectrum beta-lactamase *E. coli* World Health Organization (2021) guidelines. *tet* genes were detected using quantitative real-time polymerase chain reaction after dividing pig farms in the province into four clusters based on their adjacent areas and characteristics.

**Results:**

*tetA*, *tetB*, *tetC*, *tetM*, *tetO*, and *tetX* were detected in solid waste and wastewater from pig farms, whereas *tetE* was not detected in either sample type. *tetX* (100%) and *tetO* (75%) were the most dominant genes in solid waste, whereas wastewater samples were dominated by *tetA*, *tetM*, *tetO*, and *tetX* (prevalence of 50% each). Furthermore, eight *tet* gene patterns were found in pig farm waste (prevalence of 12.5% each).

**Conclusion:**

The results showed a high prevalence of *tetO* and *tetX* in solid waste and wastewater from pig farms in Banten province. This significant prevalence and diversity indicated the transmission of *tet* genes from pigs to the environment, posing a serious threat to public health.

## Introduction

Antibiotic resistance is a significant issue that influences the effectiveness of treatments for bacterial diseases in humans and animals, making it a major global concern [1, 2]. One factor contributing to the emergence and spread of antibiotic resistance genes (ARGs) is the long-term and extensive use of antibiotics in livestock, which has become a global challenge [3].

More than 85% of administered antibiotics or their metabolites are excreted in the urine or feces of animals and discharged into the environment [4]. The widespread use of antibiotics imposes selective pressure on bacteria, leading to resistant bacterial strains that can spread among humans, animals, and different environments [5]. Several ARGs are encoded in mobile genetic elements (MGEs), enabling their transmission when introduced into a new environment [6]. Consequently, bacteria carrying various ARGs are commonly found in livestock waste and the surrounding environment [4, 7].

Tetracyclines are commonly used in humans and livestock due to their broad-spectrum activity, availability, and affordability. These antibiotics are mostly available in healthcare units, especially community health centers (Puskesmas) [8], and they are frequently used in pig farms across Indonesia [9, 10]. Based on previous studies by Wang *et al*. [11] and Zhang *et al*. [12] various bacterial species have developed resistance to tetracycline through their *tet* genes.

Livestock feces and wastewater treated with antibiotics serve as important reservoirs of antibiotic residues, antibiotic-resistant bacteria, and ARGs that can be horizontally transferred, contributing to the emergence and spread of ARGs in the environment [13, 14]. Furthermore, livestock feces that are stored intensively, either composted or fresh, are generally used as fertilizer, leading to the contamination of agricultural land with antibiotic-resistant bacteria [14, 15]. Livestock wastewater discharged into waters and the wider terrestrial environment can contaminate water [7] and soil [14, 15], promoting the transmission of resistant bacteria and ARGs. This can cause significant environmental problems and threaten public health, particularly through contaminated food chains [16, 17].

Integrons are MGEs that facilitate gene transmission between and within species due to its location on plasmids and transposons. This phenomenon has been widely reported, especially in *Enterobacteriaceae* species [18, 19], including *Escherichia coli* [20]. *Escherichia coli* is classified as a critical pathogen and is a member of the 12 priority pathogenic families. This Gram-negative enteric commensal bacterium is commonly found in humans and animals*. Escherichia coli* is also one of the most widely used indicator organisms in monitoring antimicrobial resistance due to its susceptibility to the high selective pressure of antimicrobial agents and transmission of ARGs to other bacteria with the same or different species horizontally through MGEs or vertically through self-cleavage [21–24].

Over 40 genes encoding tetracycline resistance (*tet*) genes have been characterized. Based on their resistance mechanism, these genes were categorized as efflux pumps (n = 28), ribosomal protection proteins (n = 12), enzymatic inactivators (n = 2), and genes that induce mutations within the 16S rRNA that reduce the binding affinity of the drug for the ribosome. *tetA*, *tetB*, *tetC*, *tetD*, *tetE*, and *tetG* have been frequently associated with tetracycline resistance in *E. coli* through the efflux pump mechanism [25]. *tetM* and *tetO* detected in this study induced tetracycline resistance through ribosomal protection, whereas *tetX* is an enzymatic inactivator. Zhang *et al*. [26] detected t*etA*, *tetB*, *tetO*, and *tetE* in pig feces and farm waste. Jia *et al*. [27] also detected *tetB*, *tetC*, *tetD*, *tetE*, *tetG*, *tetL*, *tetO*, *tetM*, *tetQ*, *tetW*, *tetS*, and *tetX* in pig farm wastewater in Changzhou (Jiangsu, China).

In Indonesia, pig farms are located in Banten province to meet the demand for pork in the community, and the pig population was 7819 in 2021, according to the Banten Province Central Statistics Agency [28]. At present, there are limited data regarding the resistance to antibiotics such as tetracycline in *E. coli* and ARGs in pig farm waste in the country, especially in Banten province, highlighting the need for study in this area.

This study aimed to detect and assess the diversity of *tet* genes in *E. coli* isolated from pig farm waste in Banten province, Indonesia, to assist in developing strategies to prevent and control antimicrobial resistance.

## Materials and Methods

### Ethical approval

Ethical approval was not required for this study. However, samples were collected according to standard sampling procedures referring to SNI 6989.59–2008 [29] and ISO 19458:2006 [30].

### Study period and location

This study was conducted from July to December 2022. Isolation and identification of *E. coli* from the collected samples were performed at the Microbiology Laboratory of the School of Veterinary Medicine and Biomedical, IPB University, Indonesia. The detection of ARGs using the quantitative real-time polymerase chain reaction (qPCR) method was conducted at the Quality Control Laboratory and Certification of Animal Products, Ministry of Agriculture, Republic of Indonesia.

### Sample collection

Solid waste (feces) and wastewater samples were collected from 44 pig farms in Banten province. The solid waste was taken from a collection of fresh pig feces on farms, whereas wastewater was sampled following the standards SNI 6989.59–2008 for wastewater sampling [29] and ISO 19458:2006 regarding microbiological analysis of water quality [30]. Samples were collected aseptically and transported to the laboratory at 4°C followed by the collection of 500-mL liquid waste samples.

### Isolation and identification of *E. coli*

The isolation and identification of *E. coli* were performed following the guidelines of *Global Tricycle Surveillance* extended-spectrum beta-lactamase *E*. *coli* from the World Health Organization [31]. Serial dilutions of samples up to 1 × 10^−5^ were made in duplicate using a solution of sterile phosphate-buffered saline (PBS; pH 7.4) at a ratio of 1:9. This was followed by the collection of 0.1 mL of each dilution, which was transferred to a Petri dish containing Tryptone Bile X-Glucuronide (TBX) agar (Merck KGaA, Darmstadt, Germany) and plated on the surface of agar (spread plate method). Colonies on TBX agar suspected to be *E. coli* were round, smooth, and bluish-green. Petri dishes with ≤100 colony-forming units/mL were used in the next stage. A minimum of five colonies each selected from TBX agar were inoculated onto MacConkey agar (MCA, Oxoid, Basingstoke, UK). Morphologically, the suspected *E. coli* colonies on MCA were round, flat, smooth in shape, dark pink in color, and non-mucoid, and they were surrounded by a cloudy zone. Subsequently, the suspected *E. coli* colonies were cultured on tryptic soy agar (Oxoid) and sulfide indole motility agar (Oxoid) for the confirmatory indole biochemical test. Positive *E. coli* results were indicated by the formation of a cherry red ring, and *E. coli* ATCC 25922 served as the positive control.

### DNA extraction

DNA extraction from *E. coli* isolates was performed using a Mericon DNA Bacteria Kit (Qiagen, Hilden, Germany) according to the manufacturer’s instructions. The *E. coli* isolate was transferred using loops from the culture medium into a microtube containing 1 mL of sterile PBS until the turbidity reached at least 0.5 McFarland standard, depending on the availability of isolates. The sample was centrifuged at 13,000× *g* for 5 min, and the supernatant was discarded, leaving only the bacterial pellet. The bacterial pellets were washed by adding 200 μL of sterile PBS and homogenized using a vortex. Subsequently, the suspension was centrifuged at 13,000× *g* for 5 min, and the washing process was repeated until a colorless suspension was obtained. In total, 200 µL of Fast Lysis Buffer were added, and the suspension was heated in a ThermoMixer (Eppendorf, Hamburg, Germany) at 100°C and 122× *g* for 10 min. The suspension was incubated at room temperature (20–25°C) for 2 min and centrifuged at 13,000× *g* for 5 min. The supernatant containing DNA (100 μL) was transferred to a new 2-mL microtube and stored at −20°C or −80°C until further analysis.

The extracted *E. coli* DNA was pooled based on clusters and tested by qPCR according to these clusters. Pig farms in Banten province were divided into four clusters based on adjacent areas and characteristics. Cluster 1 (central region) consisted of one subdistrict (Neglasari) with one farm, cluster 2 (western region) comprised three subdistricts (Panongan, Legok, and Tigaraksa) with 24 farms, cluster 3 (southern region) consisted of one subdistrict (Cisauk) with two farms, and cluster 4 (northern region) comprised five subdistricts (Mauk, Teluk Naga, Paku Haji, Kosambi, and Sepatan Timur) with 17 farms.

### Quality control of DNA

The DNA concentration and purity were tested using a NanoDrop spectrophotometer (Thermo Fisher Scientific, Massachusetts, US). The DNA concentration needed for the qPCR test was >36 ng/μL, whereas the DNA purity ratio assessed by NanoDrop was commensurate with the set value of 1.8–2.0 (A260/A280).

### Detection of tetracycline resistance genes

The presence of tetracycline resistance genes was tested using the SYBR Green qPCR method and primers of the target genes listed in Table-1 [32–34]. The SYBR Green PCR method was performed on a real-time PCR thermal cycler (Rotor-Genes Q, Qiagen, Hilden, Germany). The reagents of the master mix for the SYBR Green qPCR protocol were prepared in each microtube according to the required design plate layout with the following template (25 μL): 12.5 μL of SYBR select master mix, 2 μL each of primary reverse and forward primers (10 µM), 3.5 μL of nuclease-free water, and 5 μL of the DNA sample. Each microtube was placed on a PCR plate cooler to keep the reagent at a low temperature. Subsequently, qPCR and melting were performed using Q-Rex software (Qiagen). The amplification process for *tetA*, *tetM*, *tetO*, and *tetX* followed the procedure proposed by Li *et al*. [35] using a two-step qPCR program. This involved initial heating at 95°C for 3 min, followed by 40 cycles of denaturation at 10 s 95°C, annealing for 60 s at a temperature adjusted for the primers of the target genes (as specified in Table-1), and extension for 1 min at 72°C. The amplification process using SYBR Green for *tetB*, *tetC*, and *tetE* was performed as described by Jia *et al*. [27]. The process included initial heating at 94°C for 5 min, followed by 40 cycles of denaturation for 60 s at 94°C, annealing for 30 s at a temperature selected according to the primers of the target genes (Table-1), and extension for 90 s at 72°C. The specificity of the amplified product was analyzed using a melting curve (95°C for 10 s, 65°C–95°C with a 0.5°C increase every 0.05 s).

**Table-1:** Details of the primers used to detect tetracycline resistance genes.

Gene	Primer	Primer sequence (5’ to 3’)	Temperature *Annealing* (°C)	Reference
*tetA*	*tetA*-F	GCTACATCCTGCTTGCCTTC	57	[32]
	*tetA*-R	CATAGATCGCCGTGAAGAGG		
*tetB*	*tetB*-F	TTG GTT AGG GGC AAG TTT TG	56	[32]
	*tetB*-R	GTA ATG GGC CAA TAA CAC CG		
*tetC*	*tetC*-F	CTTGAGAGCCTTCAACCCAG	55	[32]
	*tetC*-R	ATGGTCGTCATCTACCTGCC		
*tetE*	*tetE*-F	AAACCACATCCTCCATACGC	57	[32]
	*tetE*-F	AAATAGGCCACAACCGTCAG		
*tetO*	*tetO*-F	ACGGARAGTTTATTGTATACC	57	[33]
	*tetO*-R	TGGCGTATCTATAATGTTGAC		
*tetM*	*tetM*-F	ACAGAAAGCTTATTATATAAC	52	[33]
	*tetM*-R	TGGCGTGTCTATGATGTTCAC		
*tetX*	*tetX*-F	AGCCTTACCAATGGGTGTAAA	57	[34]
	*tetX*-R	TTCTTACCTTGGACATCCCG		

The results were considered positive when the cycle threshold (CT) value was <40 with an amplification curve [27, 35] and a single melt peak was formed with a melting temperature range smaller than 2°C. However, the results were considered negative/undetectable when the CT value exceeded 40 and no amplification curve was detected. When the CT value ranged >36–<40, the results were considered indeterminate/dubious.

### Statistical analysis

The data from the test results are presented in tables and figures and analyzed using a descriptive method.

## Results

### Isolation and identification of *E. coli*

All samples analyzed in this study were positive for *E. coli*, and the complete results for isolation and identification are presented in Table-2.

**Table-2:** The result of *E. coli* isolation and identification.

Sample type	Number of isolate culture	Results at each testing stages			Positive *E. coli* (%)

		TBX media culture (%)	MCA media culture (%)	Indole test (%)	
Solid waste (feces)	44	44 (100)	44 (100)	44 (100)	100
Wastewater	44	44 (100)	44 (100)	44 (100)	100

TBX=Tryptone bile X-glucuronide, *E. coli=Escherichia coli*, MCA=MacConkey agar

### Detection of tetracycline resistance genes

**Figure-1:**
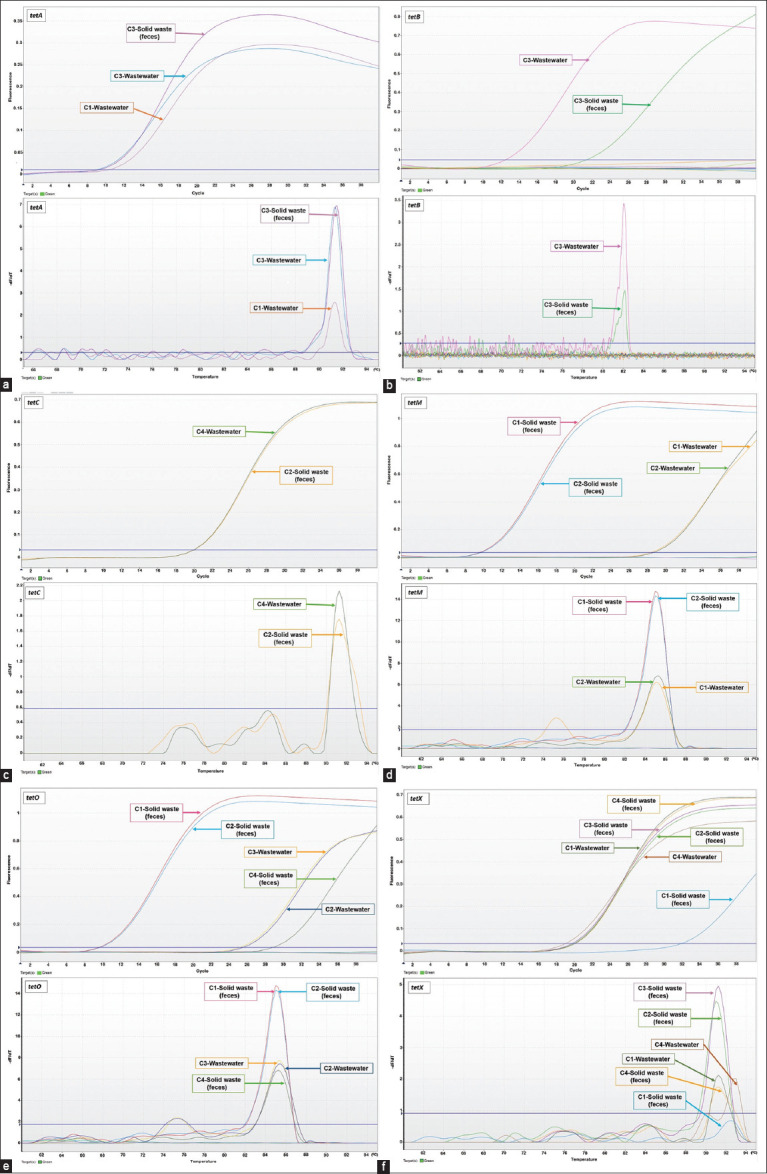
Test results showing detection of *tet* genes in solid waste (feces) and wastewater samples of pig farms by quantitative real-time polymerase chain reaction. (a) Amplification curve and melting curve of *tetA*; (b) Amplification curve and melting curve of *tetB*, (c) Amplification curve and melting curve of *tetC*, (d) Amplification curve and melting curve of *tetM*, (e) Amplification curve and melting curve of *tetO*, and (f) Amplification curve and melting curve of *tetX*.

**Figure-2:**
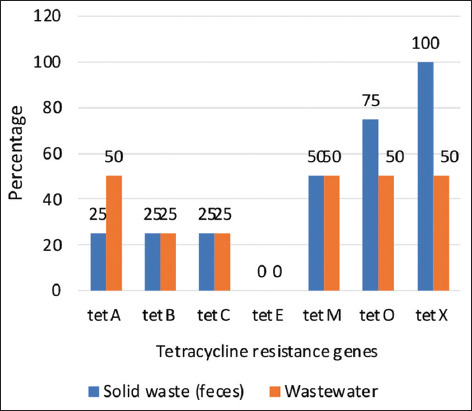
The prevalence percentage of tetracycline resistance genes in solid waste (feces) and wastewater samples from pig farms.

The assessment of tetracycline resistance genes revealed the presence of *tetA*, *tetB*, *tetC*, *tetM*, *tetO*, and *tetX* in solid waste and wastewater samples from pig farms, whereas *tetE* was not detected in either sample type (Table-3). The amplification and melting curves obtained from testing tetracycline resistance genes by qPCR are presented in Figure-1. Among the solid waste samples, *tetX* and *tetO* were the most prevalent (100% and 75%, respectively), followed by *tetM* (50%), *tetA* (25%), *tetB* (25%), and *tetC* (25%). Meanwhile, wastewater samples were dominated by *tetA*, *tetM*, *tetO*, and *tetX* (50% each), followed by *tetB* and *tetC* (25% each), as presented in Figure-2.

**Table-3:** CT and melt peak values of the *tet* genes detected in solid waste (feces) and wastewater samples of pig farms using qPCR.

Cluster and solid waste sample code	*tet* genes	CT value	Melt peak (°C)	Cluster and wastewater sample code	*tet* genes	CT value	Melt peak (°C)
Cluster 1/C1 (141A)	*tetM*	9.9	85	Cluster 1/C1 (142A)	*tetA*	11.89	90.6
	*tetO*	9.6	85		*tetM*	28.71	85.3
	*tetX*	31.72	92.5		*tetX*	19.93	91.3
Cluster 2/C2 (36C, 38D, 40D, 42C, 44D, 46A, 48A, 50B, 52B, 54B, 56A, 58B, 60A, 62B, 64D, 66E2, 68B, 70E1, 72A, 74A, 76A, 78B, 80A, 82D)	*tetC*	19.97	91.3	Cluster 2/C2 (37A, 39B, 41B, 43A, 45C, 47D2, 49A, 51B, 53A, 55A, 57A, 59A, 61A, 63B, 65A, 67A, 69D, 71B, 73A, 75B, 77A, 79A, 81B, 83A)	*tetO*	25.35	85.5
	*tetO*	9.71	85		*tetM*	28.96	85.3
	*tetM*	10.03	85				
	*tetX*	19.79	91				
Cluster 3/C3 (84A, 87A)	*tetA*	11.16	90.4	Cluster 3/C3 (85D, 88D)	*tetA*	11.1	90.5
	*tetB*	21.4	82.1		*tetB*	12.49	82
	*tetX*	19.62	91.3		*tetO*	24.96	85.3
Cluster 4/C4 (89C, 91A, 93D, 95A, 97C, 100B, 102A, 104B,106D, 108A, 110A,112A, 126A, 129A,132D, 135A, 138A)	*tetO*	28.63	85.3	Cluster 4/C4 (90E, 92A, 94A, 96A, 98E, 101D, 103A, 105D, 107A, 109A, 111A,113B, 127A, 130A, 133C, 136B, 139A)	*tetC*	19.93	91.3
	*tetX*	19.97	91.3		*tetX*	18.49	93

qPCR=Quantitative real-time polymerase chain reaction, CT=Cycle threshold

Eight *tet* gene patterns were found in pig farm samples with the same prevalence of 12.5%, as presented in Table-4.

**Table-4:** Patterns of tetracycline resistance genes in waste of pig farms samples.

*tet* genes pattern	Total number of samples	Total number (%)
*tetM* + *tetO* + *tetX*	1	12.5
*tetC* + *tetM* + *tetO* + *tetX*	1	12.5
*tetA* + *tetB* + *tetX*	1	12.5
*tetO* + *tetX*	1	12.5
*tetA* + *tetM* + *tetX*	1	12.5
*tetM* + *tetO*	1	12.5
*tetA* + *tetB* + *tetO*	1	12.5
*tetC* + *tetX*	1	12.5
Total number	8	100

## Discussion

### Isolation and identification of *E. coli*

All samples tested in this study were positive for *E. coli*, indicating a high prevalence of *E. coli* in both solid waste and wastewater from pig farms in Banten province. Similarly, the previous studies by Kallau *et al*. [36] reported a high *E. coli* prevalence of 85.40% in pig farms in Kupang City, Indonesia. This significantly high prevalence was attributable to the use of *E. coli* as a commensal bacterium and the potential of the microbe to cause various digestive tract disorders and other extra-intestinal diseases. Furthermore, this bacterium is widespread and abundant in pig farms [12], pig slaughterhouses [37], and the surrounding environment [16, 38].

The results of the survey revealed that most pig farms in Banten province were traditional or household-scale farms with pens located in close proximity to residential areas. These farms primarily raise pigs for fattening purposes, and their hygiene and sanitation were generally low. Furthermore, most of the farms did not have a waste treatment plant, leading to the direct discharge of waste into the environment. This had caused the spread of *E. coli* to aquatic and terrestrial environments, posing a serious potential threat to public health. According to Jang *et al*. [39], the presence and endurance of *E. coli* in pig feces were affected by the temperature of the environment and the hygiene and sanitation of the cage. *Escherichia coli* can survive for long periods outside the digestive tract and reproduce in soil, sand, and sediment in environments with tropical, subtropical, and warm climates [40]. Several strains of *E. coli*, including pathogenic strains such as *E. coli* O157:H7, have shown the ability to adapt to the environment and survive in fertilizer and on the surface of vegetables, namely, lettuce and spinach. The presence of pathogenic *E. coli* in food has caused outbreaks of food poisoning in the community [39].

*Escherichia coli* carries ARGs that can be transferred horizontally to bacteria of the same or different species through conjugation events such as the transfer of ARGs through plasmids or other genetic materials, namely, transposons and integrons [41]. The World Organization for Animal Health has identified *E. coli* (commensal) and *Salmonella* spp. (pathogens) as indicator bacteria in monitoring and surveillance programs of antibiotic resistance in animals and the environment. This recognition is attributable to the susceptibility of these bacteria to high selective pressure from antimicrobial agents in contact with the host, leading to an increase in the relative abundance of resistant bacterial populations [42].

### Detection of tetracycline resistance genes

In this study, seven *tet* genes responsible for the emergence of *E. coli* resistance to tetracycline antibiotics with different resistance mechanisms, including drug efflux (*tetA*, *tetB*, *tetC*, and *tetE*), ribosomal protection (*tetM* and *tetO*), and enzymatic inactivation (*tetX*), were tested. Almost all of the tested genes excluding *tetE* were detected in both solid waste and wastewater samples from pig farms. This is consistent with the findings of AbuOun *et al*. [43], who detected *tetA*, *tetB*, *tetC*, and *tetM* in pigs. Similarly, Zhang *et al*. [26] detected *tetA*, *tetB*, *tetO*, and *tetE* in pig feces and farm waste. Jia *et al*. [27] detected *tetB*, *tetC*, *tetD*, *tetE*, *tetG*, *tetL*, *tetO*, *tetM*, *tetQ*, *tetW*, *tetS*, and *tetX* in pig farm wastewater in Changzhou (Jiangsu, China).

*tetX* and *tetO* were the most common genes in the solid waste samples with prevalences of 100% and 75%, respectively, whereas the prevalence of *tetA*, *tetM*, *tetO*, and *tetX* in wastewater samples was 50%. The previous studies also reported that *tetA* was dominantly detected in pig feces (44.9% [43] and 94.7% [44]), pig farm waste (66.7% [45]), pig slaughterhouse wastewater (50% [37]), and the environment (88.9% [44] and 100% [45]). Plasmid-mediated *tetX* (variant *tetX4*) was detected in *E. coli* from samples of pig feces (31.03%), pig anal swabs (37.93%), farm environments such as water (6.89%), soil (6.89%) [46], dust (0.9%) [47], and pig slaughterhouses [48]. According to Jia *et al*. [27], *tetX* exhibited a high relative abundance in pig farm wastewater of 106.3 copies/16S rRNA gene copies. Similarly, *tetO* recorded a high relative abundance (22.71 copies/16S rDNA gene copies) in pig farm waste [26].

According to Nguyen *et al*. [25], *tetA*, *tetB*, *tetC*, *tetD*, *tetE*, and *tetG* were frequently associated with tetracycline resistance in *E. coli* through the efflux pump mechanism. *tetA* was the most dominant efflux pump gene in this study, especially in wastewater samples (50%), followed by *tetB* and *tetC* (25% each), whereas *tetE* was not detected in either sample type. This finding was consistent with those of previous studies, in which *tetA* was most commonly found in Gram-negative bacteria such as *E. coli* [20, 49]. *tetA* is located in a conjugation plasmid, facilitating the easy spread of resistance genes to other bacteria of the same or different species through horizontal gene transfer with conjugation. Similarly, *tetC* is located in bacterial plasmids, *tetB* is found on transposons and integrative and conjugative elements [50], whereas *tetE* is often associated with non-conjugative plasmids [34], limiting its transmission.

In this study, the two *tet* genes responsible for the ribosomal protection resistance mechanism were *tetO* and *tetM*. *tetO* was more prevalent than *tetM*, especially in solid waste samples. According to Avrain *et al*. [51], *tetO* is mostly associated with conjugative plasmids in *Campylobacter* spp., and transfer between *Campylobacter jejuni* isolates has been reported. Recent studies have reported that *tetO* was integrated into transposons carrying the macrolide-resistant efflux genes *mefA* and *msrD*. These transposons can be transferred conjugatively to different strains of *Streptococcus pyogenes* and unrelated *Enterococcus faecalis* [52, 53]. Roberts [54] reported that the discovery of *tetO* in conjugative transposons facilitated its wider transmission among various unrelated bacteria. Moreover, ARGs associated with conjugative transposons are more easily transmitted to other bacteria, even those that are not closely related, than non-conjugative plasmids [55]. *tetO* was rarely found in *E. coli*, but in this study, *tetO* was present at a high prevalence, especially in solid waste samples. This was attributable to the presence of *tetO* in conjugative transposons and plasmids, making it possible for a wider horizontal transmission to other unrelated bacteria, such as *E. coli*.

*tetM* genes have been detected in enterococci [56] and are related to transposons and conjugative plasmids [57, 58], facilitating the transmission of resistance genes to other bacteria. Although *tetM* has rarely been detected in Gram-negative coliforms such as *E. coli*, this study recorded a fairly high prevalence of 50% in solid waste and wastewater samples from pig farms. This was in line with previous findings in which *tetM* was detected in 13.1% of tetracycline-resistant *E. coli* isolates from ileal samples from healthy pigs [59]. The fairly high prevalence of *tetM* in this study was related to the horizontal transfer of genes from bacteria in the digestive tract of pigs, such as enterococci to *E. coli* to a process involving transposons and plasmid conjugation.

In this study, only *tetX* utilized the enzymatic inactivation resistance mechanism, and it was present in 100% of solid waste samples. This finding can be explained by the fact that *tetX* in *E. coli* can be located in plasmids, which are highly transferable and successfully mobilized in *Enterobacteriaceae* bacteria. The *tetX* variant *tetX4* is most commonly found in mobile plasmids, enabling the sharing of genetic information among different bacteria [47]. Furthermore, this gene has been identified in *E. coli*, but the resistance mechanisms of enzymatic inactivation have rarely been described by Poirel *et al*. [20]. According to Zhang *et al*. [60], plasmid-mediated *tetX* (*tetX4*) was detected in isolates from various animals, including pigs, ducks, geese, chickens, cattle, freshwater fish, and shrimp, as well as migratory birds, with pigs being the predominant source. Li *et al*. [46] isolated 32 *tetX4*-positive strains from pig feces and anal swabs in Shanxi, China. Similarly, *tetX4*-positive *E. coli* was detected in the sewage and soil of pig farms. These isolates had different ST types, but their *tetX4*-carrying plasmids comprised the same replicon type. This indicated that the plasmids were transferred horizontally among different reservoirs, leading to *tetX4* transmission in the surrounding environment. Several other studies have detected *tetX* in pig feces [61], farm wastewater [62], well water around farms [63], and river water [64].

The *tet* gene patterns formed in this study comprised at least two or four *tet* gene combinations. Specifically, eight *tet* gene patterns were found in pig farm samples at the same prevalence of 12.5%. The highest diversity was found in cluster 1 in solid waste samples, which featured a combination of four *tet* genes (*tetC*, *tetO*, *tetM*, and *tetX*), followed by three-type combinations in cluster 1 in solid waste (*tetO*, *tetM*, and *tetX*) and wastewater samples (*tetA*, *tetM*, and *tetX*) and in cluster 3 in solid waste (*tetA*, *tetB*, and *tetX*) and wastewater samples (*tetA*, *tetB*, and *tetO*). This high diversity indicated the spread of *tet* genes from pig farms to the environment due to the excessive and uncontrolled use of tetracycline antibiotics in farms. Tetracycline antibiotics such as oxytetracycline and tetracycline have been frequently used in Indonesian pig farms for both therapeutic and nontherapeutic purposes (prophylactic, metaphylactic) as well as for growth promotion [9, 65, 66]. According to Kallau *et al*. [9], 55.21% of antibiotics in pig farms were used for treatment, whereas 42.71% and 2.08% were used for disease prevention and production enhancement, respectively.

Pig feces and farm waste are important reservoirs of antibiotic-resistant bacteria and ARGs. Horizontal gene transfer involving MGEs plays a crucial role in the formation, dissemination, and assembly of various ARGs among different bacterial cells, resulting in the combination and diversity of these genes [13, 14]. Plasmids also play a significant role in transferring multidrug resistance genes between bacterial species and closely related different species [67]. Furthermore, integrons found in plasmids and/or transposons significantly contribute to the increasing transmission of ARGs. Class 1 integrons are mostly involved in the dissemination of ARGs in Gram-negative and-positive bacteria [68]. Moredo *et al*. [69] reported that approximately 17.5% of *E. coli* isolates in pig farms carried integrons as propagators of antibiotic resistance to the environment. *Escherichia coli* strains resistant to multiple tetracyclines can increase the possibility of combinations or new *tet* gene patterns. The occurrence of *tet* gene combinations is a serious problem with a significant impact on human health and the environment [70].

The occurrence of antibiotic-resistant *E. coli* in pigs poses a significant risk, as it can lead to the contamination of pork [66] and processed meat products [71], as well as aquatic [27] and terrestrial environments [72] through improper handling of waste generated. Based on the field survey, most pig farms in Banten province were close to community settlements, and the generated waste was not handled properly. This condition created a high risk of contaminating the environment and the wider ecosystem with antibiotic-resistant bacteria and ARGs, leading to serious effects on public health through the contaminated food chain.

## Conclusion

*tetX* and *tetO* were the most dominant tetracycline resistance genes found in waste from pig farms in Banten province. The presence of eight *tet* gene patterns at the same prevalence suggested a high prevalence and diversity of *tet* genes in the waste sample. This indicated the transmission of *tet* from pigs to the environment had occurred, posing a serious threat to public health.

## Authors’ Contributions

DFP: Conducted the study, sample and data collection, sample testing, data analysis, and drafted the manuscript. HL and IWTW: Conducted the study, interpretation of the data, and drafted and revised the manuscript. CB and PR: Conducted the study, data analysis, and manuscript preparation. All authors have read, reviewed, and approved the final manuscript.
